# Isolation of Phages for Phage Therapy: A Comparison of Spot Tests and Efficiency of Plating Analyses for Determination of Host Range and Efficacy

**DOI:** 10.1371/journal.pone.0118557

**Published:** 2015-03-11

**Authors:** Mohammadali Khan Mirzaei, Anders S. Nilsson

**Affiliations:** Department of Molecular Biosciences, The Wenner-Gren Institute, Stockholm University, Stockholm, Sweden; Rockefeller University, UNITED STATES

## Abstract

Phage therapy, treating bacterial infections with bacteriophages, could be a future alternative to antibiotic treatment of bacterial infections. There are, however, several problems to be solved, mainly associated to the biology of phages, the interaction between phages and their bacterial hosts, but also to the vast variation of pathogenic bacteria which implies that large numbers of different phages are going to be needed. All of these phages must under present regulation of medical products undergo extensive clinical testing before they can be applied. It will consequently be of great economic importance that effective and versatile phages are selected and collected into phage libraries, i.e., the selection must be carried out in a way that it results in highly virulent phages with broad host ranges. We have isolated phages using the *Escherichia coli* reference (ECOR) collection and compared two methods, spot testing and efficiency of plating (EOP), which are frequently used to identify phages suitable for phage therapy. The analyses of the differences between the two methods show that spot tests often overestimate both the overall virulence and the host range and that the results are not correlated to the results of EOP assays. The conclusion is that single dilution spot tests cannot be used for identification and selection of phages to a phage library and should be replaced by EOP assays. The difference between the two methods can be caused by many factors. We have analysed if the differences and lack of correlation could be caused by lysis from without, bacteriocins in the phage lysate, or by the presence of prophages harbouring genes coding for phage resistance systems in the genomes of the bacteria in the ECOR collection.

## Introduction

Phage therapy involves clinical treatment of bacterial infections with phages (bacteriophages). The method, which has gained a renewed interest because of increasing frequency of infections by multidrug-resistant bacteria, has potential benefits, but much research remains before the method is fully developed and can be considered an important contribution to the fight against severe bacterial infections [1–8]. The present limitations of phage therapy depend mainly on two causes: Firstly, on the large natural variation of phages and target bacteria, and secondly on the regulatory framework for clinical application of phages. The large variation of increasingly multiresistant bacteria causing infections will demand a large number of different and well-characterised phages that all have been approved for clinical treatment. This can be accomplished by the establishment of phage libraries consisting of phages that all have passed clinical trials. The libraries would constantly be complemented with new phages, which of course also need to have passed clinical trials, as new bacterial strains appear. When establishing a phage library, it is highly desirable that collected phages show high virulence on a large number of bacterial strains. Selecting phages with a wide host range limits the number of phages in the library, and reduces the cost for clinical trials. It would also increase the probability that a phage in the library could be used against emerging multiresistant bacterial strains. Selecting phages that are highly virulent, having a high burst size (i.e. producing a large number of progeny phage), is equally important. Phages are huge compared to small antibiotic molecules. They cannot be administered in high doses and thus diffuse very poorly [2]. A high burst size increases the probability that phages reach target bacteria, which is crucial for achieving a productive infection, and reaching the benefits that phages have compared with antibiotics [9]. If phages can eliminate infecting bacteria faster than they can replicate, a high burst size also results in a lower risk of selection for phage resistant bacteria [9].

The host range and burst size of phages are hence two important parameters when phages are to be selected for phage libraries, and for efficient and effective phage therapy. The first step in the isolation of new phages to a library is usually to amplify the phages present in a sample from nature using cultures of target bacteria by infecting each of the strains in a well-defined bacteria collection. This is usually done in liquid culture by mixing a larger volume of the sample with the same volume of double concentrated medium and bacteria from an overnight culture. This causes a selection of phages which are fast, i.e. have a short latency period, if these are initially more prevalent than other phages in the sample. This may be desirable from a phage therapy point of view, but a phage with long latency period and higher burst size can be lost, or rather never found, if a quick phage is present in sufficiently high titre in the sample from the beginning, even if this rapid phage has only a fraction of the slower phage’s burst size.

In the following step, the isolated phages are characterized. In addition to determining the latency period and burst size from infection of the primary host bacterium [10], phages are also tested for the ability to grow on other hosts for assessment of host range, usually against the strains in the collection of bacteria used for isolation, but it could also be done against any other varied collection. This is often accomplished with a spot test, which means to examine on which bacterial strains a particular phage produces plaques [11,12]. Small droplets of the phage lysate are either stamped or trickled on a plate prepared with the bacterial strain to be tested. Sometimes different concentrations of phage lysate are used in the estimation of host range in this way, but often only one concentration is used, extracted and applied directly from the lysate of the primary host bacterium. Spotting several dilutions onto the bacteria is better than using just one high titre lysate [12]. At a low concentration of phages, the number of phages infecting a cell is reduced. A PubMed search with the search string “phage isolation AND host range” resulted in 13 articles published in 2014. Nine of these papers report the application of either single dilution spot tests, where the phage titre was either not mentioned or varied substantially, or by a poorly described method [13–21]. It is not uncommon for individual phages to express a host range of around half of the bacteria of a large bacterial collection used for the screening as a result of a spot test, which can sometimes result in a host range of 30–40 strains of bacteria per phage and also include bacteria from other genera than the phages were isolated from. A phage infection is however a complicated process and a positive spot test, commonly observed clear zones on a bacterial plate infected with phages, may be the result of more than the interactions which give rise to lysis and production of new phage. The lysate might contain residues of a bacteriocin which kills bacteria, and phages themselves can give rise to either abortive infection or lysis from without, both forming clear zones on a bacterial plate without new phages being produced [9]. Host range must be defined based on the number of hosts on which a particular phage gives rise to a productive infection, i.e. a production of progeny phages. Furthermore, the productive infections of a phage on different strains ought to result in burst sizes comparable to the outcome of infecting its primary bacterial host, in order for the phage to be selected for a phage therapy library, but burst sizes are difficult to estimate from the result of spot tests. The concept of host range is however not clear but can be defined in many different ways. The method used should be accounted for when assessments of host ranges are reported as a basis for selecting phages for phage therapy, since it reflects what is actually measured [9]. The efficiency of plating (EOP) differs from the spot test in this respect as it measures the titre of phage progeny from a particular phage infecting a bacterial strain [9,12]. Of the 13 reviewed papers only one followed up initial spot tests with EOP analyses [22], and another three made serial dilution spot tests [23–25].

We have used the ECOR collection [26] for isolation of virulent phages suitable for phage therapy. The initial spot tests, of all isolated phages against all ECOR strains, showed that there were many candidate phages, and the six phages exhibiting the widest host range were selected for further estimation of host range by EOP within ECOR as well as within the Salmonella collections SARA [27] and SARB [28], and a collection of clinical isolates of ESBL-producing *Escherichia coli*. We have compared the observed host range estimated in spot test assays to the EOP, and discuss in this article the importance of the different methods for selecting phages for phage therapy.

## Materials and Methods

### Bacteria

The ECOR standard reference collection of *E*. *coli* (72 strains) [26]; the two *Salmonella* reference collections SARA (72 strains) [27], and SARB (70 strains, i.e. two strains missing) [28]; and an additional 20 clinical isolates of ESBL-carrying *E*. *coli* (collected by the Public Health Agency of Sweden) were used. Bacterial cultures were grown at 30°C overnight (18 hours) to reach stationary phase, and assure a similar concentration of bacteria in the comparative studies of spot tests and efficiency of plating. For the enrichment of phages, the bacterial cultures used were grown at 37°C on a shaker incubator for 4–6 hours.

### Culturing media

All bacteria were grown in lysogeny broth (LB) media or on lysogeny broth agar (LA) plates [29], except the ESBL-carrying bacteria where the media was supplemented with 100 μg/ml ampicillin. Soft agar (SA), made from 0.6% LB, was used for top agar in the plaque assays.

### Bacteriophages

The bacteriophages were isolated from sewage water sampled at the Käppala waste water treatment plant (location: WGS84: 59°21&rsquo;22.2"N 18°13&rsquo;45.3"E), recipient of waste water from Stockholm city including some hospitals, and kept at +4°C before processing. The sampling of water was approved by the owner, Käppalaförbundet, Box 3095, 181 03 Lidingö, Sweden, kappala@kappala.se (Phages are not considered to be protected species). The phages were amplified by mixing 50 ml of waste water with the same amount of double strength LB and 10 ml of a single ECOR strain bacteria cultured overnight. After incubation overnight at 30°C, 10 ml of the mixture was shaken with 1% v/v chloroform and left at room temperature for 30 minutes to kill the bacteria, centrifuged at 3000×g at +4°C for 15 minutes, and sterile filtered through a 0.45 μm membrane filter (Whatman, ref. no. 10462100). After checking the lysates for phages, the titre was measured in plaque assays. Sterile filtered phage lysates were diluted in SM buffer [30] or LB to five different dilutions (10^-5^–10^-9^). 100 μl of diluted phage and 200 μl of target bacteria were mixed with 2 ml SA, spread on pre-warmed LA plates, and incubated overnight at 30°C [31]. The harvested phages were selected according to their plaque morphology. Phages displaying large, clear and non-turbid plaques without a halo were classified as virulent. Phages were re-isolated by plaque purification from the LA plates when several phages on the same plate could be suspected. After additional plaque purifications, 25 virulent phages were saved and stored in 50% glycerol at -70°C as well as in LB at +4°C [32]. These phages were named according to guidelines in Kropinski et al. [33]. The six phages showing the broadest host range in the spot test (see below) were thus named as follows; vB_EcoP_SU10, vB_EcoP_SU16, vB_EcoP_SU27, vB_EcoP_SU32, vB_EcoP_SU57, and vB_EcoP_SU63, but only the last part of names is used in the following text, e.g. SU10, SU16 and so on.

### Degree of adsorption

The host bacterium was grown to mid-exponential phase and phages added at a concentration approximately equivalent to a multiplicity of infection (MOI) of five. After five minutes incubation at 37°C, the culture was put on ice, 1% chloroform added, and subsequently centrifuged for 10 minutes at +4°C. The supernatant was sterile filtered and the PFU was measured. The degree of adsorption was calculated as the number of adsorbed phages in five minutes by subtracting the remaining phage concentration from the starting titre [10].

### Latency period and burst size

The host bacterium was grown overnight and a high titre phage stock was prepared for the determination of latency period and burst size. 100 μl of overnight cultured bacteria was added to 20 ml fresh media and grown at 37°C on a shaking incubator to mid-exponential phase. A sample of the bacterial culture was taken for titre determination immediately before phages were added, again at a concentration roughly equivalent to a MOI of five, in order to assure that only a small fraction of bacteria would not get infected [11]. The number of unadsorbed phages was calculated after five minutes by a plaque assay of a sample of the phage infected culture. The bacterial optical density (OD) was measured every five minutes, to the first drop at which the culture was sampled to calculate the PFU of the phage, at the time of burst. The latency period was calculated as the difference between time of inoculation and the time for the first drop in OD. The burst size was calculated as the number of free phages, measured as PFU in the plaque assay, minus the unadsorbed phages divided by the initial bacterial titre.

### Spot assay

200 μl of target bacteria suspension incubated overnight was spread on LA plates and incubated for 40 minutes at 30°C. Phages were spotted onto the surface of the plates at a titre of the order 10^9^ phages/ml with either a cross test comb or a pipette both holding 10 μl of individual phage lysates. The plates were left to dry and were inspected for lysis zones after an overnight incubation at 30°C. The spot assay was used to assess the bactericide ability of all of the 25 isolated virulent phages to form clear zones on the bacterial strains of the ECOR collection, and was repeated three times for each phage. Six phages (see above) were also selected for spot assays on the strains of the other tree collections, SARA, SARB and ESBL-producing *E*. *coli*, and for the following EOP assays on the ECOR collection.

### Efficiency of plating

The six phages that displayed the widest bactericide host range in the spot assays were selected for a more thorough assessment of productive infection as defined by the efficiency of plating (EOP). Each phage was tested three times for each of four different dilutions against all the bacterial strains that it had been shown to be able to lyse in the spot assays. This was done under the same conditions as in the spot assays, i.e. using stationary phase bacteria. Thus, all bacterial strains to be tested were grown overnight (18 hours) at 30°C and 200 μl of each of those cultures was used in double layer plaque assays together with 100 μl of diluted phage lysate. The four phage lysates were 10^6^–10^9^ times dilutions from the phage stock. This means that EOP assay replicates for a particular phage were done in parallel on all bacterial strains tested, and also at concentrations comparable to what was used in the spot tests. The plates were incubated overnight at 30°C and the number of plaque forming units (PFU) was counted for each combination. When the 10^6^ dilution did not result in any plaques, a lower dilution was tried afterwards to verify that the EOP was lower than 0.001. Finally, the EOP was calculated (average PFU on target bacteria / average PFU on host bacteria) along with the standard deviation for the three measurements (S1 Table).

The average EOP value for a particular phage—bacterium combination was classified as “High production” when the ratio was 0.5 or more, i.e. when the productive infection on the target bacterium resulted in at least 50% of the PFU found for the primary host. An EOP of 0.1 or better, but below 0.5, was considered to be of “Medium production” efficiency, and between 0.001 and 0.1 as “Low production” efficiency. An EOP equal to or under 0.001 was classified as inefficient [34].

### Transmission electron microscopy (TEM)

10 μl of a phage lysate with a high titre was spotted onto carbon coated grids and stained with 1% uranyl acetate. The negatively stained grids were observed in a Tecnai G2 transmission electron microscope at 80kV.

## Results and Discussion

**Fig 1:**
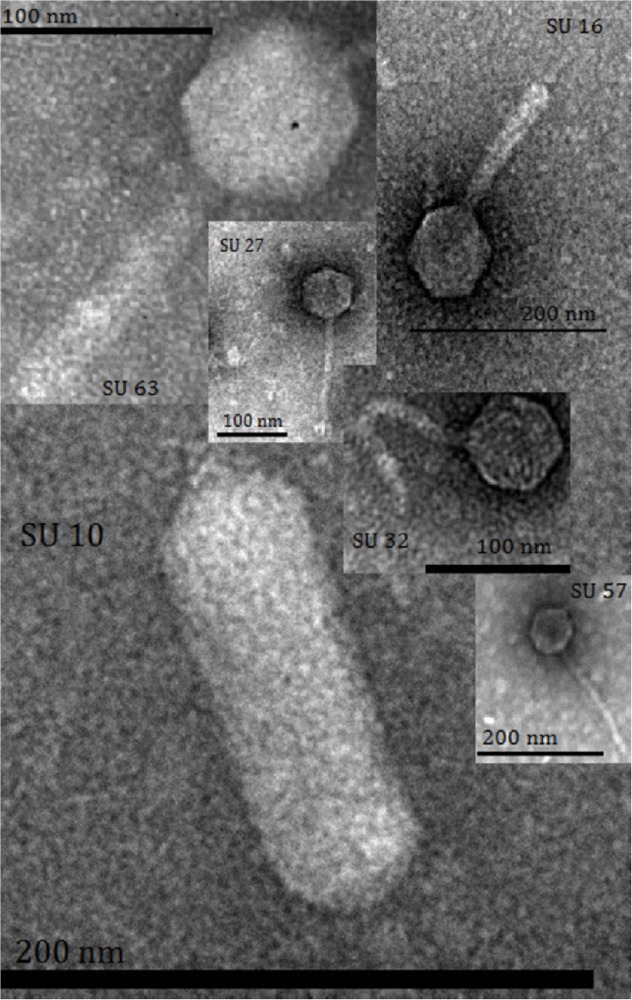
Transmission electron micrographs of the six phages in this study.

Six phages were selected as they were found to have remarkably wide host ranges in spot test screens of 25 phages individually spotted onto each strain of the ECOR collection. The 25 phages had previously been classified as virulent based on their ability to produce large and clear plaques in plaque assays. The initial characterization of morphology by TEM revealed that the six phages were from all three families within the *Caudovirales* order (Table 1). The sizes of phages were calculated from the images, and showed that one phage, SU10, was approximately 137 nm long and of the quite unusual C3 morphotype (Fig. 1; Table 1) [35]. SU10 was shown to have the longest latency period, 47 minutes, and a burst size of 166. C3 phages have previously been shown to possess large genomes and lower virulence, with a latency period between 30–40 minutes, a burst size between 100–144, and poor adsorption rate [36,37]. The virulence of the other five phages was more varied as the latency periods were found to be 17–45 minutes, and the burst sizes 109–196 PFU/cell (Table 1).

**Table 1:** Taxonomy, morphology and virulence data of the six bacteriophages.

Bacteriophage	SU10	SU16	SU27	SU32	SU57	SU63
Family	*Podoviridae*	*Myoviridae*	*Siphoviridae*	*Siphoviridae*	*Siphoviridae*	*Myoviridae*
Morphotype^a^	C3	A1	B1	B1	B1	A1
Capsid size, nm ± SE	137 ± 4.2 × 57 ± 3.5	71 ± 0.7	69 ± 0.7	58 ± 2	59 ± 2.8	72 ± 0.5
Tail length, nm ±SE	19 ± 1.5	127 ± 0.7	164 ± 6.3	154 ± 6.3	168 ± 4	119 ± 7
Latency period, min	47	43	37	33	17	45
Burst size, PFU/cell	166	175	137	148	196	109
Adsorption degree^b^, %	98	94	90	93	90	97

^a^ See reference [47].

^b^ Phage particles adsorbed within 5 min.

The spot test assays of the six phage lysates showed that host ranges varied as well. The phage SU57 had the capacity to lyse 15% (11 strains) whereas the SU16 lysate caused lysis zones on around 60% (43 strains) of the 72 strains of the ECOR collection (Table 2). The EOP analyses, as expected, showed another picture. The proportion of strains where high productive infection (EOP ≥ 0.5) was achieved was considerably lower than the results of the spot tests and varied between 1%, or 1 out of the 72 strains for the SU57 phage, and 14% (10 strains) for the SU10 phage (Table 2; S1 Table). On average, a high productive infection (EOP ≥ 0.5) was only evident from around 17% of the strains (4.83 compared to 28.33) that had shown sensitivity in the spot tests, and the SU16 infection resulted in a high productive infection in only eight cases compared with the 43 lysed strains in the spot test (Table 2). Even if all EOP classes were considered (i.e. High + Medium + Low) the average number of strains producing phages upon infection was still only around half of the result from the spot test. There could be many reasons for this [9], but colicins cannot be one of them. All strains in the ECOR collection have been typed for colicin encoding plasmids, and it is only the lysate of ECOR63 that may contain a colicin, of the E1 type, and only one of the other ECOR strains are sensitive to that type of colicin [38]. Lysis from without is another plausible mechanism which happens when an overload of phages simultaneously infects a bacterium leading to lysis either from the action of lysins or from rapid depletion of the cells resources [39]. However, this requires quite a high MOI, in the range of 100-fold more phages than bacteria, and is thus an unlikely explanation to the difference between the spot tests and the EOP results. High EOP was in addition not correlated to the results from the spot tests. The correlation coefficient for the phages’ number of positive spot tests (first row in Table 2) and the number of high EOP strains (second row in Table 2) was 0.46 (P = 0.29). The correlation coefficient was still not significant even if lower EOPs were included as well. Including the number of strains with an EOP down to 20% of the efficacy on the host strain results in a correlation coefficient of 0.68 (P = 0.14) to the corresponding number of positive spot tests strains (Table 2; S1 Table). The phage titre is also important for reaching a productive infection during phage therapy, but the titre of individual phages would in a phage cocktail get lower and lower as more phages are added. A phage with narrow host range and/or low efficacy must thus be discarded when assembling a phage cocktail. The result from additional spot tests showed that a cocktail of the six phages lysed 55 of the 72 strains in the ECOR collection, but the EOP data showed that if the criteria above would have been applied, selecting only phages with high EOP (≥ 0.5) for a cocktail, the cocktail would theoretically have been useful against only 17 strains (S1 Table).

**Table 2:** Results of spot test assays and efficiency of plating (EOP) on strains of the ECOR collection.

Bacteriophage	SU10	SU16	SU27	SU32	SU57	SU63	Average	%
Number of ECOR strains (n = 72)								
Lysed strains in spot test assays^a^	30	43	32	18	11	36	28.33	39
High production, (EOP ≥ 0.5)	10	8	1	5	1	4	4.83	7
Medium production, (0.1≤ EOP <0.5)	2	8	3	0	1	5	3.17	4
Low production, (0.001< EOP <0.1)	10	12	15	4	1	11	8.83	12
No production, (EOP ≤ 0.001)	8	15	13	9	8	16	11.50	16
High EOP /spot test ratio	0.33	0.19	0.03	0.28	0.09	0.11	0.17	-
High+Medium/spot test ratio	0.40	0.37	0.13	0.28	0.18	0.25	0.27	-
Sum of EOP values	8.68	9.37	2.30	10.12	1.32	4.08	5.81	-

^a^ The correlation analyses were done on the six phages’ data in this row against the data in the second row or against the sum of the data in the second and following rows.

The distribution of EOP into different classes of efficacy also revealed that there are many phage—bacterium combinations that result either in low EOP (>0.001 – <0.1) or occasional plaques hidden in the no plating class (≤ 0.001). The distribution of EOPs seems bimodal, with too few medium EOP values. Apparently, if the effect of colicins and lysis from without can be ruled out, phages probably inject their DNA but the infection cycle halts at some later stage. If there would have been a strong correlation between spot test and EOP results it would have implied a general system that inhibited the phages, at a later stage in their life cycle, and in a systematic way, but the poor correlation between lysing ability and efficacy shows that there is no such mechanism. Part of the reason behind the lack of correlation is instead probably that the main part of bacterial resistance against phages is not “adsorption resistance” but internal resistance that is transcriptional dependent. Many phages can attach and infect (inject DNA), because the surface variation is limited, but encounter various resistance mechanisms genetically encoded by the host. R/M, CRISPR-cas, Abi, and various defence genes carried by plasmids or prophages, are all transcription dependent systems [9]. There is a transcriptional race between the phage early genes, for establishing its own independent machinery, and the host´s defence system. Occasionally, a phage can succeed in producing offspring although the genome of the bacterium harbours genetic resistance systems. This could lead to a few plaques being observed, cause a non-systematic random pattern, and the lack of correlation between spot testing and EOP. Phage SU10 had the highest EOP /spot test ratio (0.33) among the six phages. It also has a large genome of 77 kb [35]. A hypothesis that remains to be tested is that phages with large genomes contain genes necessary for overcoming “internal” resistance by inhibiting or interfering with genetically controlled resistance systems encoded by the host. The SU57 phage, in contrast, had only an EOP / spot test ratio of 0.09 since a high EOP was only reached on one strain, the primary isolation strain. Interestingly, SU10 is also the slowest phage and SU57 the fastest, a fact that together with the host range could illustrate the theory of trade-offs in phages´ life histories [40]. Slow phages with low virulence are supposed to be generalists with a wide host range, found in nutrient poor environments where hosts are scarce whereas highly virulent phages are expected to be specialists with a narrow host range and found where bacterial densities are high. In our data, the correlation coefficient between the number of strains where high EOP was achieved and virulence calculated as 1/latency period in minutes × burst size was-0.47. Although not strongly correlated, there was a tendency for phages with low virulence to have a wider host range.

Apart from well-known bacterial resistance systems against phages, resistance genes can also be carried by prophages. For instance, P2 is a temperate phage that can form rather stable lysogens, it is not inducible with either UV or mitomycin [41,42], and it has been shown that it carries genes at least at three loci that bring resistance to the host bacterium. P2 has the ability to convert its host to become resistant against T4, T5, and λ [43–45]. We have showed that there is substantial variation at these loci implying that other P2-like prophages could bring resistance to other virulent phages [46]. We have also previously investigated the presence of P2-like phages among the strains of the ECOR collection, and 20 of the 72 strains were shown to harbour a complete P2-like phage and another 6 at least large parts of its genome [46] (S1 Table). The high frequency and stability of P2-like prophages in *E*. *coli* genomes implies an important role for these phages in the defence against virulent phage attacks. In this study, we investigated whether the presence / absence of a P2-like phage affected the probability to find infections from highly virulent phages by checking if P2-like prophages were present in the genomes of the bacteria that had showed high sensitivity (EOP ≥ 0.5) as well as in low sensitivity (EOP < 0.5) strains (S1 Table). Although the difference was not statistically significant (Fisher’s exact test, P = 0.065), there is a tendency that higher EOPs are more unlikely to be found when infecting strains contain a P2-like prophage. In addition, of the 29 phages that produced a high EOP (≥ 0.5), only three did it on strains containing a complete or almost complete P2-like prophage, as judged from the hybridisation signals against a probe consisting of whole genome DNA from phage P2 where the two strongest signal categories (3 and 4 out of four) indicated a complete or almost complete prophage genome (S1 Table). Thus, the capability of prophages to inhibit the propagation of virulent phages is something that definitely needs more attention in future phage therapy research.

The six selected phages were also tested against ESBL expressing strains of *E*. *coli*, initially in spot tests where 16 of the 20 multiresistant strains were shown to be sensitive to one or more phage lysates. The EOP of the six phages on these 16 strains showed results comparable to the result of the EOP assays on the ECOR collection; a high efficacy (EOP ≥ 0.5) was only achieved in 12% of the individual cases where a phage was tested on a multiresistant strain (Table 3), but several phages were effective against the same bacterial strain resulting in an overall efficacy against seven out of the 20 bacterial strains (S1 Table). On the other hand, the EOP was higher than on the original strain of isolation for eight phage—bacterium combinations. This demonstrates the relative ease to isolate new and highly effective phages against these strains that cause severe infections which are very hard to cure. A cocktail of these six phages would in theory be effective against a large number of ESBL carrying *E*. *coli* strains.

**Table 3:** Results of spot test assays and efficiency of plating (EOP) on ESBL-*E*. *coli*, SARA, and SARB strains.

Bacteriophage	SU10	SU16	SU27	SU32	SU57	SU63	Average	%
Number of ESBL strains (n = 20)								
Lysed strains in spot test assays	7	9	13	7	2	9	7.83	39
High production, (EOP ≥ 0.5)	2	5	4	1	1	1	2.33	12
Medium production, (0.1≤ EOP <0.5)	0	0	0	0	0	0	0.00	0
Low production, (0.001< EOP <0.1)	2	0	1	0	0	0	0.50	3
No production, (EOP ≤ 0.001)	3	4	8	6	1	8	5.00	25
Number of SARA strains (n = 72)								
Lysed strains in spot test assays	2	1	2	7	3	1	2.67	4
High production, (EOP ≥ 0.5)	0	0	0	0	0	0	0.00	0
Medium production, (0.1≤ EOP <0.5)	0	0	0	2	1	0	0.50	1
Low production, (0.001< EOP <0.1)	0	0	0	0	0	0	0.00	0
No production, (EOP ≤ 0.001)	2	1	2	5	2	1	2.17	3
Number of SARB strains (n = 70)								
Lysed strains in spot test assays	3	2	1	5	4	4	3.17	16
High production, (EOP ≥ 0.5)	0	0	0	0	1	0	0.17	1
Medium production, (0.1≤ EOP <0.5)	0	0	0	1	0	0	0.17	1
Low production, (0.001< EOP <0.1)	0	0	0	1	0	0	0.17	1
No production, (EOP ≤ 0.001)	3	2	1	3	3	4	2.67	13
High EOP /spot test ratio	0.17	0.42	0.25	0.05	0.22	0.07	0.20	-
High+Medium/spot test ratio	0.17	0.42	0.25	0.21	0.33	0.07	0.24	-
Sum of EOP values	1.94	5.85	3.96	2.67	3.30	1.30	3.17	-

The spot tests indicated that several phages were capable of causing bacterial killing of many *Salmonella* strains as well; i.e. nine SARA strains and 11 SARB strains were lysed when the six phages were applied. A collection of phages with a host range covering bacteria from more than one genus would facilitate the compilation of phage libraries and reduce the number of necessary phage stocks for phage therapy. There are reports of phages being effective against more than one bacterial species [9], but the EOP assays on the nine SARA strains revealed that productive infection was only achieved on two bacterial strains of the 72 strains in the collection (S1 Table). In addition, the EOP was under the 50% limit in both of these strains. The six phages had an equally low EOP against the 11 SARB strains that had been lysed in spot tests. Only one phage could be infected, but surprisingly that one phage had an EOP exceeding the EOP on its *E*. *coli* host by 30% (Table 3; S1 Table).

## Conclusion

Isolation of phages for phage therapy will in the future never be “on demand” because of the need for proper testing and approval for clinical use. There is an excess of phages to be found against every bacterial strain but only highly virulent phages with a broad host range will be selected. The reason for this is more or less simply economical. The phages would have to be tested one by one, and the cost for a single clinical trial is very expensive. High virulence depends on the burst size of a phage, which is a prerequisite for reaching productive infection which in turn is of key importance for successful treatment. The selection of phages can however not be based on spot tests which merely reflects a bactericidal effect. Spot test results of burst sizes and host ranges are not correlated to results of high efficiency of plating (EOP), which is a better method that should consequently be applied in the selection process. EOP analyses also show that the initial isolation of phages often results in phages with a higher efficacy on other bacteria than the primary strain of isolation. Taken together, this shows the need for better isolation methods, which takes into account that phages with longer latency period, or phages with a low titre in a sample, may have a higher burst size.

The single round of isolation of phages using the ECOR collection resulted in phages that were able to lyse, and had a high EOP, on several ESBL *E*. *coli* strains. The six phages could potentially be used against seven (35%) of the 20 ESBL strains, which shows that it is possible to use a collection of non-pathogenic bacteria for isolation of phages that are equally or even more virulent on other bacteria (Table 3; S1 Table). It is desirable however to include as many bacterial strains as possible in the screening panel, and to perform EOP assays on all phages. It is quite possible that there were phages among the 25 initially selected phages that possibly would have had a high EOP on several of the 20 ESBL *E*. *coli* strains.

There is also a need for a better knowledge about encoded bacterial resistance systems, their prevalence and genetic variation, and establishment of collections of bacteria containing as large part of this variation as possible. In this paper, we have pointed out that more studies on the importance of resistance genes, against virulent phage attacks, carried by prophages are needed. The *E*. *coli* phage P2 is a well-spread phage that conveys resistance against several virulent phages, and at the same time is quite stable as a prophage. Our results indicate that *E*. *coli* lysogens are harder to infect when they harbour a P2-like prophage, but also that more data is needed to corroborate this.

## Supporting Information

S1 Table Efficiency of plating (EOP) for the six bacteriophages on bacterial hosts from the ECOR, SARA, and SARB collections and a collection of clinical ESBL *E*. *coli* isolates. (DOCX)

## References

[1] Verbeken G, Pirnay JP, Lavigne R, Jennes S, De Vos D, Casteels M, et al Call for a dedicated European legal framework for bacteriophage therapy. Arch Immunol Ther Exp (Warsz). 2014;62: 117–129. 10.1007/s00005-014-0269-y 24500660 PMC3950567

[2] Nilsson AS. Phage Therapy—Constraints and Possibilities. Ups J Med Sci. 2014;119: 192–198. 10.3109/03009734.2014.902878 24678769 PMC4034558

[3] Kutter E, De Vos D, Gvasalia G, Alavidze Z, Gogkhia L, Kuhl S, et al Phage therapy in clinical practice: Treatment of human infections. Curr Pharm Biotechnol. 2010;11: 69–86. 20214609 10.2174/138920110790725401

[4] Abedon ST, Kuhl SJ, Blasdel BG, Kutter EM. Phage treatment of human infections. Bacteriophage 2011;1: 66–85. 22334863 10.4161/bact.1.2.15845 PMC3278644

[5] Pirnay JP, De Vos D, Verbeken G, Merabishvili M, Chanishvili N, Vaneechoutte M, et al The phage therapy paradigm: Pret-a-porter or sur-mesure? Pharm Res 2011;28: 934–937. 10.1007/s11095-010-0313-5 21063753

[6] Abedon ST, Thomas-Abedon C. Phage therapy pharmacology. Curr Pharm Biotechnol. 2010;11: 28–47. 20214606 10.2174/138920110790725410

[7] Brüssow H. Phage Therapy: Quo vadis? Clin Infect Dis. 2014;58: 535–536. 10.1093/cid/cit776 24270168

[8] Gill J, Hyman P. Phage choice, isolation, and preparation for phage therapy. Curr Pharm Biotechnol. 2010;11: 2–14. 20214604 10.2174/138920110790725311

[9] Hyman P, Abedon ST. Bacteriophage host range and bacterial resistance. Adv Appl Microbiol. 2010;70: 217–248. 10.1016/S0065-2164(10)70007-1 20359459

[10] Hyman P, Abedon ST. Practical methods for determining phage growth parameters. Methods Mol Biol. 2009;501: 175–202. 10.1007/978-1-60327-164-6_18 19066822

[11] Carlson K. Working with bacteriophages: Common techniques and methodological approaches In: Kutter E, Sulakvelidze A, editors. Bacteriophages: Biology and Application. Boca Raton, FL, USA: CRC Press; 2005 pp. 437–494.

[12] Kutter E. Phage host range and efficiency of plating. Methods Mol Biol. 2009;501: 141–149. 10.1007/978-1-60327-164-6_14 19066818

[13] Tsonos J, Oosterik LH, Tuntufye HN, Klumpp J, Butaye P, De Greve H, et al A cocktail of in vitro efficient phages is not a guarantee for in vivo therapeutic results against avian colibacillosis. Vet Microbiol. 2013;171: 470–479. 10.1016/j.vetmic.2013.10.021 24269008

[14] Wongsuntornpoj S, Moreno Switt AI, Bergholz P, Wiedmann M, Chaturongakul S. Salmonella phages isolated from dairy farms in Thailand show wider host range than a comparable set of phages isolated from U.S. dairy farms. Vet Microbiol. 2014;172: 345–352. 10.1016/j.vetmic.2014.05.023 24939592 PMC4157059

[15] Frampton RA, Taylor C, Holguin Moreno AV, Visnovsky SB, Petty NK, Pitman AR, et al Identification of bacteriophages for biocontrol of the kiwifruit canker phytopathogen Pseudomonas syringae pv. actinidiae. Appl Environ Microbiol. 2014;80: 2216–2228. 10.1128/AEM.00062-14 24487530 PMC3993152

[16] Abbasifar R, Kropinski AM, Sabour PM, Chambers JR, MacKinnon J, Malig T, et al Efficiency of bacteriophage therapy against Cronobacter sakazakii in Galleria mellonella (greater wax moth) larvae. Arch Virol. 2014;159: 2253–2261. 10.1007/s00705-014-2055-x 24705602

[17] Al-Fendi A, Shueb RH, Ravichandran M, Yean CY. Isolation and characterization of lytic vibriophage against Vibrio cholerae O1 from environmental water samples in Kelantan, Malaysia. J Basic Microbiol. 2014;54: 1036–1043. 10.1002/jobm.201300458 24532381

[18] Di Lallo G, Evangelisti M, Mancuso F, Ferrante P, Marcelletti S, Tinari A, et al Isolation and partial characterization of bacteriophages infecting Pseudomonas syringae pv. actinidiae, causal agent of kiwifruit bacterial canker. J Basic Microbiol. 2014;54: 1210–1221. 10.1002/jobm.201300951 24810619

[19] Cavanagh D, Guinane CM, Neve H, Coffey A, Ross RP, Fitzgerald GF, et al Phages of non-dairy lactococci: isolation and characterization of PhiL47, a phage infecting the grass isolate Lactococcus lactis ssp. cremoris DPC6860. Front Microbiol. 2014; 4: 417 10.3389/fmicb.2013.00417 24454309 PMC3888941

[20] Dziewit L, Oscik K, Bartosik D, Radlinska M. Molecular characterization of a novel temperate sinorhizobium bacteriophage, ΦLM21, encoding DNA methyltransferase with CcrM-like specificity. J Virol 2014;88: 13111–13124. 10.1128/JVI.01875-14 25187538 PMC4249059

[21] Bourdin G, Navarro A, Sarker SA, Pittet AC, Qadri F, Sultana S, et al Coverage of diarrhoea-associated Escherichia coli isolates from different origins with two types of phage cocktails. Microb Biotechnol. 2014;7: 165–176. 10.1111/1751-7915.12113 24528873 PMC3937720

[22] Silva YJ, Costa L, Pereira C, Mateus C, Cunha A, Calado R, et al Phage therapy as an approach to prevent Vibrio anguillarum infections in fish larvae production. PLoS One 2014;9: e114197 10.1371/journal.pone.0114197 25464504 PMC4252102

[23] Kim M, Kim S, Park B, Ryu S. Core lipopolysaccharide-specific phage SSU5 as an Auxiliary Component of a Phage Cocktail for Salmonella biocontrol. Appl Environ Microbiol. 2014;80: 1026–1034. 10.1128/AEM.03494-13 24271179 PMC3911222

[24] Castillo D, Christiansen RH, Espejo R, Middelboe M. Diversity and geographical distribution of Flavobacterium psychrophilum isolates and their phages: patterns of susceptibility to phage infection and phage host range. Microb Ecol. 2014; 67: 748–757. 10.1007/s00248-014-0375-8 24557506

[25] Wittmann J, Dreiseikelmann B, Rohde C, Rohde M, Sikorski J. Isolation and characterization of numerous novel phages targeting diverse strains of the ubiquitous and opportunistic pathogen Achromobacter xylosoxidans. PLoS One 2014;9: e86935 10.1371/journal.pone.0086935 24466294 PMC3899368

[26] Ochman H, Selander RK. Standard reference strains of Escherichia coli from natural populations. J Bacteriol. 1984;157: 690–693. 6363394 10.1128/jb.157.2.690-693.1984 PMC215307

[27] Beltran P, Plock SA, Smith NH, Whittam TS, Old DC, Selander RK. Reference collection of strains of the Salmonella typhimurium complex from natural populations. J Gen Microbiol. 1991;137(Pt 3): 601–606. 2033380 10.1099/00221287-137-3-601

[28] Boyd EF, Wang FS, Beltran P, Plock SA, Nelson K, Selander RK. Salmonella reference collection B (SARB): strains of 37 serovars of subspecies I. J Gen Microbiol. 1993;139(Pt 6): 1125–1132. 8360609 10.1099/00221287-139-6-1125

[29] Bertani G. Studies on lysogenesis I. The mode of phage liberation by lysogenic Escherichia coli. J Bacteriol. 1951;62: 293–300. 14888646 10.1128/jb.62.3.293-300.1951 PMC386127

[30] Sambrook J, Russell DW. Molecular cloning—A laboratory manual. New York: Cold Spring Harbor Laboratory Press; 2001.

[31] Pieroni P, Rennie RP, Ziola B, Deneer HG. The use of bacteriophages to differentiate serologically cross-reactive isolates of Klebsiella pneumoniae. J Med Microbiol. 1994;41: 423–429. 7966220 10.1099/00222615-41-6-423

[32] Clark WA. Comparison of several methods for preserving bacteriophages. Appl Microbiol. 1962;10: 466–471. 14021544 10.1128/am.10.5.466-471.1962 PMC1057894

[33] Kropinski AM, Prangishvili D, Lavigne R. Position paper: the creation of a rational scheme for the nomenclature of viruses of Bacteria and Archaea. Environ Microbiol. 2009;11: 2775–2777. 10.1111/j.1462-2920.2009.01970.x 19519870

[34] Viazis S, Akhtar M, Feirtag J, Brabban AD, Diez-Gonzalez F. Isolation and characterization of lytic bacteriophages against enterohaemorrhagic Escherichia coli. Appl Microbiol. 2011;110:1323–1331. 10.1111/j.1365-2672.2011.04989.x 21362115

[35] Khan Mirzaei M, Eriksson H, Kasuga K, Haggård-Ljungquist E, Nilsson AS. Genomic, proteomic, morphological, and phylogenetic analyses of vB_EcoP_SU10, a Podoviridae phage with C3 morphology. PLoS One 2014;9: e116294 10.1371/journal.pone.0116294 25551446 PMC4281155

[36] Kleppen HP, Holo H, Jeon SR, Nes IF, Yoon SS. Novel podoviridae family bacteriophage infecting Weissella cibaria isolated from kimchi. Appl Environ Microb. 2012;78: 7299–7308. 22885743 10.1128/AEM.00031-12 PMC3457107

[37] Matsushita K, Uchiyama J, Kato S, Ujihara T, Hoshiba H, Sugihara S, et al Morphological and genetic analysis of three bacteriophages of Serratia marcescens isolated from environmental water. FEMS Microbiol Lett. 2009;291: 201–208. 10.1111/j.1574-6968.2008.01455.x 19087204

[38] Riley M, Gordon DM. A survey of col plasmids in natural isolates of Escherichia coli and an investigation into the stability of col-plasmid lineages. J Gen Microbiol. 1992;138: 1345–1352. 1512564 10.1099/00221287-138-7-1345

[39] Abedon ST. Lysis from without. Bacteriophage 2011;1: 47–49. 10.4161/bact.1.1.13980 PMC3109453 21687534

[40] Keen EC. Tradeoffs in bacteriophage life histories. Bacteriophage 4: e28365 24616839 10.4161/bact.28365 PMC3942329

[41] Bertani LE, Bertani G. Genetics of P2 and related phages. Adv Genet. 1971;16: 199–237. 4947104 10.1016/s0065-2660(08)60359-4

[42] Nilsson AS, Haggård-Ljungquist E. The P2-like bacteriophages In: Calendar R, editor. The Bacteriophages. New York: Oxford University Press; 2006 pp. 365–390.

[43] Calendar R, Yu S, Myung H, Barreiro V, Odegrip R, Carlson K, et al The lysogenic conversion genes of coliphage P2 have unusually high AT content In: Syvanen M, Kado CI, editors. Horizontal Gene Transfer. London: Chapman & Hall; 1998 pp. 241–252.

[44] Mosig G, Yu S, Myung H, Haggård-Ljungquist E, Davenport L, Carlson K, et al A novel mechanism of virus-virus interactions: Bacteriophage P2 Tin protein inhibits phage T4 DNA synthesis by poisoning the T4 single-stranded DNA binding protein, gp32. Virology 1997;230: 72–81. 9126263 10.1006/viro.1997.8464

[45] Odegrip R, Nilsson AS, Haggard-Ljungquist E. Identification of a gene encoding a functional reverse transcriptase within a highly variable locus in the P2-like coliphages. J Bacteriol. 2006;188: 1643–1647. 16452449 10.1128/JB.188.4.1643-1647.2006 PMC1367236

[46] Nilsson AS, Karlsson JL, Haggård-Ljungquist E. Site-specific recombination links the evolution of P2-like coliphages and pathogenic enterobacteria. Mol Biol Evol. 2004;21: 1–13. 12949155 10.1093/molbev/msg223

[47] Ackermann HW. Frequency of morphological phage descriptions in the year 2000. Brief review. Arch Virol. 2001;146: 843–857. 11448025 10.1007/s007050170120

