# Correction: Isolation of Phages for Phage Therapy: A Comparison of Spot Tests and Efficiency of Plating Analyses for Determination of Host Range and Efficacy

**DOI:** 10.1371/journal.pone.0127606

**Published:** 2015-05-07

**Authors:** Mohammadali Khan Mirzaei, Anders S. Nilsson

In Table 3, the ESBL, SARA, SARB should be under separated headline. Please see the corrected Table 3 here.

**Table 3:** Results of spot test assays and efficiency of plating (EOP) on ESBL-*E*. *coli*, SARA, and SARB strains.

Bacteriophage	SU10	SU16	SU27	SU32	SU57	SU63	Average	%
Number of ESBL strains (n = 20)								
Lysed strains in spot test assays	7	9	13	7	2	9	7.83	39
High production, (EOP ≥ 0.5)	2	5	4	1	1	1	2.33	12
Medium production, (0.1≤ EOP <0.5)	0	0	0	0	0	0	0.00	0
Low production, (0.001< EOP <0.1)	2	0	1	0	0	0	0.50	3
No production, (EOP ≤ 0.001)	3	4	8	6	1	8	5.00	25
Number of SARA strains (n = 72)								
Lysed strains in spot test assays	2	1	2	7	3	1	2.67	4
High production, (EOP ≥ 0.5)	0	0	0	0	0	0	0.00	0
Medium production, (0.1≤ EOP <0.5)	0	0	0	2	1	0	0.50	1
Low production, (0.001< EOP <0.1)	0	0	0	0	0	0	0.00	0
No production, (EOP ≤ 0.001)	2	1	2	5	2	1	2.17	3
Number of SARB strains (n = 70)								
Lysed strains in spot test assays	3	2	1	5	4	4	3.17	16
High production, (EOP ≥ 0.5)	0	0	0	0	1	0	0.17	1
Medium production, (0.1≤ EOP <0.5)	0	0	0	1	0	0	0.17	1
Low production, (0.001< EOP <0.1)	0	0	0	1	0	0	0.17	1
No production, (EOP ≤ 0.001)	3	2	1	3	3	4	2.67	13
High EOP /spot test ratio	0.17	0.42	0.25	0.05	0.22	0.07	0.20	-
High+Medium/spot test ratio	0.17	0.42	0.25	0.21	0.33	0.07	0.24	-
Sum of EOP values	1.94	5.85	3.96	2.67	3.30	1.30	3.17	-
