# Supplementary material for: Isolation of Phages for Phage Therapy: A Comparison of Spot Tests and Efficiency of Plating Analyses for Determination of Host Range and Efficacy
Source: PLoS One. 2015 Mar 11;10(3):e0118557. doi: 10.1371/journal.pone.0118557 (PMC4356574; doi:10.1371/journal.pone.0118557)
Supplement: S1 Table — (DOCX) [file pone.0118557.s001.docx]

**S1 Table.** Efficiency of plating (EOP) for the six bacteriophages on bacterial hosts from the ECOR, SARA, and SARB collections and a collection of clinical ESBL *E. coli* isolates. Each EOP value is the mean of three measurements, followed by its standard deviation. The plating on the original strain of isolation (EOP = 1.0) is marked in bold.

| Bacteriophage | SU10 | SU16 | SU27 | SU32 | SU57 | SU63 |  |  |
| --- | --- | --- | --- | --- | --- | --- | --- | --- |
| ECOR |  |  |  |  |  |  | Col  plasmid^c^ | P2 prophage^d^ |
| 1 |  |  |  |  |  |  |  | - |
| 2 |  |  |  |  |  |  |  | - |
| 3 |  |  |  |  |  |  | Ia | - |
| 4 | < 0.001^a^ | 0.04±0.02 | < 0.001 | 0 |  | < 0.001 |  | 4 |
| 5 |  |  | 0 |  |  |  |  | 3 |
| 6 |  |  |  |  | < 0.001 |  |  | - |
| 7 |  |  |  |  |  |  |  | 3 |
| 8 |  |  |  |  |  | 0 |  | - |
| 9 |  |  |  |  |  |  |  | - |
| 10 | **1.0** | 0.006±0.003 | 0 |  |  | 0.25±0.22 |  | 2 |
| 11 |  |  |  |  |  |  | E(E2) | - |
| 12 |  |  |  |  |  |  | E(E1) | 3 |
| 13 | 0^b^ |  | < 0.001 |  |  |  |  | 1 |
| 14 |  |  |  |  |  |  | Ia | 1 |
| 15 |  |  |  | 0.003±0.002 |  |  | Ia | 2 |
| 16 |  | **1.0** | 0.4±0.2 | 0 |  | 0.11±0.06 |  | - |
| 17 |  |  |  |  |  |  |  | 1 |
| 18 |  |  |  |  |  |  |  | - |
| 19 |  | 0 |  |  |  |  |  | - |
| 20 |  | < 0.001 | < 0.001 |  |  |  |  | - |
| 21 |  | < 0.001 |  |  |  |  |  | - |
| 22 |  |  | < 0.001 |  |  | < 0.001 |  | - |
| 23 | 0.76±0.07 | 0.10±0.01 | < 0.001 | < 0.001 |  | 0.004±0.002 |  | - |
| 24 | < 0.001 |  |  |  |  |  | E(E1) | - |
| 25 |  |  |  |  |  |  | B | - |
| 26 |  |  | 0.24±0.02 | 0.0012±0.0002 |  | < 0.001 |  | 1 |
| 27 |  | 0.55±0.06 | **1.0** | 0.005±0.005 |  | < 0.001 |  | - |
| 28 |  |  |  |  |  |  | Ia | - |
| 29 |  | < 0.001 | 0.001 |  |  | 0 |  | - |
| 30 |  | < 0.001 | 0.015±0.003 |  |  | < 0.001 |  | 4 |
| 31 |  |  |  |  |  |  | E(E1) | 2 |
| 32 | 0.90±0.08 | 1.1±0.3 | 0.006±0.001 | **1.0** |  |  |  | - |
| 33 | 0.068±0.001 | 0.011±0.008 | 0.002±0.001 |  |  |  |  | - |
| 34 |  | < 0.001 | 0 |  |  | 0.008±0.005 | Ia | - |
| 35 | 0.008±0.001 | 0.013±0.005 |  | < 0.001 |  | 0.13±0.14 |  | - |
| 36 | 0.60±0.08 | 0.22±0.15 |  | < 0.001 | < 0.001 | 0.05±0.04 | E(E1) | - |
| 37 |  |  |  |  |  | < 0.001 |  | - |
| 38 | < 0.001 | < 0.001 |  |  |  | < 0.001 | Ia | 1 |
| 39 | < 0.001 | 0 |  |  |  |  | E(E1) | 1 |
| 40 | 0.91±0.04 | 0.14±0.03 | < 0.001 |  | < 0.001 | 0.11±0.01 | E(E1) | - |
| 41 | 0.078±0.009 | 0.05±0.03 |  |  | < 0.001 |  | K / N | - |
| 42 | < 0.001 | 0.03±0.01 |  |  |  | < 0.001 | B | 1 |
| 43 |  | < 0.001 |  |  |  | < 0.001 |  | - |
| 44 |  |  |  |  |  |  |  | 3 |
| 45 |  |  |  |  |  |  |  | 4 |
| 46 |  | < 0.001 |  |  |  |  |  | 4 |
| 47 | 0.042±0.007 | 0.008±0.003 | 0.020±0.004 |  |  |  |  | - |
| 48 | 0.35±0.09 | 0.04±0.02 | 0.005±0.002 |  |  | 0.08±0.06 | Ib | 3 |
| 49 | 0.074±0.009 | 0.03±0.01 | 0.001±0.001 |  |  |  |  | - |
| 50 | 0.026±0.003 | 0.011±0.008 |  |  | < 0.001 | 0.002±0.001 | E(E1) | 1 |
| 51 | 0.78±0.03 | 0.20±0.06 | 0.007±0.001 | 1.56±0.58 | < 0.001 | 0.5±0.3 |  | - |
| 52 | 0.07±0.01 | 0.4±0.2 | < 0.001 | < 0.001 |  | 0.09±0.07 |  | - |
| 53 |  | < 0.001 | 0 | 1.7±0.6 |  | 0.070±0.074 |  | 3 |
| 54 | 0.73±0.12 | 0.18±0.09 | 0.015±0.003 | 1.24±0.06 |  | 0.067±0.046 |  | - |
| 55 | 0.09±0.01 | 1.02±0.20 | 0.003±0.001 | 3.8±2.3 |  | 0.0153±0.0005 | ? | - |
| 56 | 0.86±0.02 | 1.0±0.2 | 0.009±0.008 |  |  | 0.63±0.15 |  | - |
| 57 | 0.72±0.12 | 1.2±0.1 | 0.006±0.003 | < 0.001 | **1.0** | 0.3±0.2 |  | - |
| 58 |  |  |  |  |  |  |  | 4 |
| 59 | 0.39±0.05 | 0.53±0.06 | < 0.001 |  |  | 0 |  | 3 |
| 60 |  | < 0.001 |  | 0.004±0.002 |  |  | E(E2) | - |
| 61 | < 0.001 | 0.07±0.01 | 0.012±0.016 | < 0.001 | 0.011±0.009 | 0.5±0.3 | Ib | 3 |
| 62 | 0.57±0.07 | 1.0±0.4 |  |  | < 0.001 | 0.015±0.018 | B | 2 |
| 63 |  | 0.03±0.01 | < 0.001 |  | 0.3±0.1 | **1.0** | E(E1) | - |
| 64 | 0.58±0.12 | 0.9±0.2 |  |  | < 0.001 |  |  | 2 |
| 65 |  | < 0.001 |  |  |  |  |  | 1 |
| 66 |  |  | 0.015±0.005 | 1.8±0.5 |  | 1.06±0.85 | ? | - |
| 67 | 0 | < 0.001 | 0.21±0.04 |  |  | < 0.001 |  | 4 |
| 68 | 0.07±0.01 | 0.14±0.01 | 0.021±0.003 | < 0.001 |  | 0.08±0.07 |  | 1 |
| 69 |  |  |  |  | 0 |  |  | - |
| 70 |  |  |  |  |  |  |  | - |
| 71 | 0.003±0.001 | 0.34±0.04 | 1.3±0.2 |  |  | < 0.001 | E(E1) | - |
| 72 |  | < 0.001 |  |  |  | < 0.001 |  | - |
|  |  |  |  |  |  |  |  |  |
|  |  |  |  |  |  |  |  |  |
| ESBL *E. coli* |  |  |  |  |  |  |  |  |
| 19 | 0.04±0.02 |  | 0 | 0 |  | 0 |  |  |
| 22 |  |  |  |  |  |  |  |  |
| 32 | 0.9±0.3 | 0.6±0.3 | 0.06±0.03 |  |  | < 0.001 |  |  |
| 39 | 1.0±0.3 | 1.6±0.2 |  |  | 0 |  |  |  |
| 41 |  | 0.95±0.33 |  |  |  | 0 |  |  |
| 44 | 0.003±0.001 | 1.2±0.1 | 1.1±0.4 | 0.001±0.0008 |  | < 0.001 |  |  |
| 45 |  |  |  |  |  |  |  |  |
| 84 |  |  |  |  |  |  |  |  |
| 127 |  |  | 0.9±0.3 | 0.001±0.0004 |  |  |  |  |
| 128 |  | 1.5±0.4 | 0.8±0.2 | < 0.001 |  | < 0.001 |  |  |
| 130 |  |  | < 0.001 | < 0.001 |  | 0 |  |  |
| 133 |  | 0 |  |  |  | 0 |  |  |
| 134 |  |  | 0 | 0 |  |  |  |  |
| 165 |  | < 0.001 | 0 |  |  |  |  |  |
| 170 |  |  | < 0.001 |  |  |  |  |  |
| 198 | 0 |  | 1.1±0.4 | 1.55±0.35 | 1.6±0.4 | 1.3±0.1 |  |  |
| 232 |  |  | 0 |  |  |  |  |  |
| 314A | 0 | 0 | 0 |  |  | 0 |  |  |
| 314B | 0 | 0 | 0 |  |  |  |  |  |
| 345 |  |  |  |  |  |  |  |  |
|  |  |  |  |  |  |  |  |  |
|  |  |  |  |  |  |  |  |  |
| SARA |  |  |  |  |  |  |  |  |
| 15 |  |  |  | 0.47±0.03 | 0.4±0.1 | 0 |  |  |
| 17 |  |  |  | 0 |  |  |  |  |
| 32 | 0 |  |  |  |  |  |  |  |
| 33 |  |  | 0 |  | 0 |  |  |  |
| 34 |  |  |  | 0 |  |  |  |  |
| 42 |  |  |  | 0 |  |  |  |  |
| 45 | 0 | 0 | 0 | 0 | 0 |  |  |  |
| 50 |  |  |  | 0.34±0.09 |  |  |  |  |
| 61 |  |  |  | 0 |  |  |  |  |
|  |  |  |  |  |  |  |  |  |
|  |  |  |  |  |  |  |  |  |
| SARB |  |  |  |  |  |  |  |  |
| 4 |  |  |  | 0.004±0.001 | < 0.001 |  |  |  |
| 5 |  | 0 |  |  |  |  |  |  |
| 13 |  |  | 0 | 0 |  |  |  |  |
| 21 | 0 |  |  | 0.3±0.005 | 1.3±0.5 |  |  |  |
| 47 | 0 |  |  |  |  | 0 |  |  |
| 48 | 0 |  |  | 0 | 0 |  |  |  |
| 49 |  |  |  |  |  | 0 |  |  |
| 50 |  | 0 |  |  |  |  |  |  |
| 51 |  |  |  | 0 | < 0.001 |  |  |  |
| 57 |  |  |  |  |  | 0 |  |  |
| 67 |  |  |  |  |  | < 0.001 |  |  |

^a^ A value < 0.001 indicates that the PFU of the actual phage on the target bacterium is more than 1000 times less than on the primary host.

^b^ A value of 0 means that bacteria only did show sensitivity in a spot test.

^c^ Data from [32].

^d^ Data from [40]. A dash signifies no hybridisation signal against a whole genome P2 probe, 1 – 4 are different degrees of hybridisation signals, where 4 denotes the strongest.
